# Associations of stable psychological traits with multi-omic subtypes of Alzheimer’s dementia

**DOI:** 10.1038/s41398-026-04195-z

**Published:** 2026-06-21

**Authors:** Andrea R. Zammit, Lei Yu, Victoria N. Poole, Shinya Tasaki, Ricardo Vialle, Konstantinos Arfanakis, Julie A. Schneider, Vladislav A. Petyuk, Philip L. De Jager, Rima Kaddurah-Daouk, Yasser Iturria-Medina, David A. Bennett

**Affiliations:** 1 Rush Alzheimer’s Disease Center, Rush University Medical Center, Chicago, IL USA; 2 Department of Psychiatry and Behavioral Sciences, Rush University Medical Center, Chicago, IL USA; 3 Department of Neurological Sciences, Rush University Medical Center, Chicago, IL USA; 4 Department of Orthopedic Surgery, Rush University Medical Center, Chicago, IL USA; 5 Department of Diagnostic Radiology and Nuclear Medicine, Rush University Medical Center, Chicago, IL USA; 6 Department of Biomedical Engineering, Illinois Institute of Technology, Rush University Medical Center, Chicago, IL USA; 7 Department of Pathology, Rush University Medical Center, Chicago, IL USA; 8 Biological Sciences Division, Pacific Northwest National Laboratory, Richland, WA USA; 9 Center for Translational & Computational Neuroimmunology, Department of Neurology, Columbia University Medical Center, New York, NY USA; 10 Department of Psychiatry and Behavioral Sciences, Duke University, Durham, NC USA; 11 Duke Institute of Brain Sciences, Duke University, Durham, NC USA; 12 Department of Medicine, Duke University, Durham, NC USA; 13 Neurology and Neurosurgery Department, Montreal Neurological Institute, Montreal, QC Canada; 14 McConnell Brain Imaging Centre, Montreal Neurological Institute, Montreal, QC Canada; 15 Ludmer Centre for Neuroinformatics & Mental Health, Montreal, QC Canada; 16 McGill University Research Centre for Studies in Aging, Douglas Research Centre, Montreal, QC Canada

**Keywords:** Molecular neuroscience, Psychiatric disorders

## Abstract

Psychological traits reflecting neuroticism, depressive symptoms, loneliness, and purpose in life are risk factors of AD dementia; however, the underlying biological mechanisms remain largely unknown. Using multi-omic data from the dorsolateral prefrontal cortex of 822 decedents in the Religious Orders Study and Rush Memory and Aging Project, we utilized a previously derived multi-omic brain molecular pseudotime representing molecular distance from no cognitive impairment (NCI) to AD dementia, and three distinct multi-omic brain molecular subtypes of AD dementia. We first confirmed generalizability of pseudotime and subtypes in two independent samples. We then annotated the subtypes, and explored whether they differed by neuropathologic burden, brain morphology or genetic risk, and found that while these indices differentiated all subtypes from NCI they did not differentiate amongst them. Finally, we tested for differential associations between the psychological traits and the subtypes, adjusting first for age, sex, education, and time to death, and then additionally for 9 common AD and Related Dementias pathologies. We found that in fully adjusted models, neuroticism, loneliness and purpose in life remained differentially associated with some AD subtypes relative to NCI. Our results are consistent with a two-stage model in which (i) upstream genetic risk influences overall disease liability, while (ii) intermediary psychological predispositions align more directly with subtype differentiation capturing AD-related heterogeneity not explained by neuropathology or brain atrophy. These results indicate that psychological risk factors may be associated with AD dementia via multi-omic molecular pathways, predominantly informed by metabolomic dysregulation, capturing heterogeneity not explained by neuropathology.

## Introduction

Psychological traits, such as neuroticism, depressive symptoms, loneliness, and purpose in life, are robust predictors of cognitive decline, incident mild cognitive impairment (MCI) and Alzheimer’s disease and related dementias (AD/ADRD) [1–5], and are linked to morbidity and mortality [6–11]. Their public health significance is well-documented [12–16].

In our cohorts, these traits predicted cognitive decline, and incident MCI and AD/ADRD independent of common age-related neuropathologic indices [17–24]. We showed limited or null associations between these traits and common markers of neurodegeneration [25]; however, in-vivo biomarker studies documented mixed evidence [26–31]. Collectively, these findings suggest that psychological-related pathways to AD/ADRD risk may not act solely through AD pathophysiology, but might instead influence broader molecular processes [32, 33].

We recently integrated four layers of dorsolateral prefrontal cortex (DLPFC) omics (epigenomic, transcriptomic, proteomic, and metabolomic) data with multimodal contrastive Trajectories Inference (mcTI) analysis to derive multi-omic pseudotime from no cognitive impairment (NCI) to AD dementia [34]. AD pseudotime is an increasingly commonly used putative time-series reconstruction technique that orders cross-sectional, typically high-dimensional data along a continuum to model molecular progression of AD disease from a low-risk to a high-risk state [35–37]. Since not everyone follows the same molecular sequence of AD disease progression, we subsequently decomposed pseudotime into three multi-omic brain molecular subtypes representing three distinct molecular pathways from NCI to AD dementia [34].

Here, we (i) first evaluated the portability of pseudotime and the subtypes to two independent samples, (ii) annotated subtype composition and top features, (iii) tested whether the subtypes differed by neuropathologic burden, brain morphology or genetic risk, and finally we (iv) tested whether long-standing traits – neuroticism, depressive symptoms, loneliness and/or purpose in life would be differentially associated with these AD dementia molecular pathways. Associations would provide clues to intermediary molecular pathways that are related both to AD dementia and its long-standing risk-factors.

## Subjects and methods

### Study population

Participants were community-based older adults from the Religious Orders Study (ROS) or the Rush Memory and Aging Project (MAP) [38]. ROS, initiated in 1994, includes older priests, nuns, and brothers from across the U.S. while MAP established in 1997, includes older men and women from across the greater Chicago metropolitan area. Participants were free of known dementia at enrollment.

### Ethics approval and consent to participate

All methods were performed in accordance with the relevant guidelines and regulations. All participants signed an informed consent, an Anatomic Gift Act to donate their brains at death and a repository consent allowing their data to be repurposed. Both studies were approved by an Institutional Review Board of Rush University Medical Center.

### Brain multi-omic data and pre-established brain multi-omic molecular AD subtypes

ROSMAP brain multi-omic data contain DNA methylation (DNAm) with Illumina 450 array, bulk next generation RNA sequencing, targeted protein expression with selected reaction monitoring, and metabolite level (metabolon) from DLPFC, generated as described previously [39–41]. We previously used the machine-learning mcTI algorithm to generate a brain molecular pseudotime of AD dementia relative to NCI, and three brain molecular subtypes reflecting different molecular pathways from NCI to AD dementia based on the numbers and types of omics [34].

### Clinical diagnoses

AD dementia was diagnosed by an experienced clinician using criteria of the joint working group of the National Institute of Neurologic and Communicative Disorders/Stroke/AD and Related Disorders Association [42], as previously described [43]. These criteria require a history of cognitive decline and evidence of impairment in at least two domains of cognitive function, one of which must be memory [44]. MCI required evidence of impairment without meeting accepted criteria for dementia, and NCI refers to individuals without dementia or MCI as previously established [44, 45].

### Neuropathologic evaluation

Brain autopsy followed standardize protocols [46, 47]. Neuropathologic evaluations systematically assessed common AD and non-AD neurodegenerative and cerebrovascular conditions including Alzheimer’s disease pathology, Lewy bodies, transactive response DNA binding protein (TDP)-43, hippocampal sclerosis, chronic macroscopic and microinfarcts, cerebral amyloid angiopathy(CAA), atherosclerosis, and arteriolosclerosis, as described in more detail in the eMethods.

### Postmortem Imaging

A subset of 278 participants underwent a postmortem brain imaging protocol, described previously [48]. Briefly, after one month postmortem, the cerebral hemisphere selected for neuropathological examination was imaged with a multi-echo spin-echo sequence on one of four 3-Tesla MRI scanners, and the resulting images were used for deformation-based morphometry(DBM) as detailed in eMethods.

### Polygenic risk scores

We utilized the PGS Catalog Calculator [49] v2.0.0 to calculate polygenic scores (PGS) for ROSMAP individuals with genotyping data. The automated pipeline obtains the scoring files from the PGS Catalog API and applies liftover if necessary. We set the genome build to GRCh38, and a minimum percentage of variant overlap was set to 50%. Additionally, no multi-allelic or ambiguous matching variants were allowed. PGS were normalized by ancestry using the HDGP and 1kGP as reference panels. As a result, 802 pre-calculated PGS covering 244 phenotypes and diseases including various cardiovascular diseases and cancers were obtained (S.Table 1).

### Assessment of psychological risk factors

Neuroticism was assessed using either 12 or 6 items from the NEO Five-Factor Inventory, which was administered at baseline or near baseline as previously reported [50]. We also used baseline assessments of depressive symptoms which were assessed using a 10-item form [51] of the Center of Epidemiologic Study-Depression Scale(CES-D) [52]; and loneliness and purpose in which were assessed in MAP participants only, using a 5-item version from a modified scale of the de Jong-Gierveld Loneliness scale [53, 54] and a 10-item scale derived from Ryff’s Scales of Psychological Wellbeing as previously described [55, 56] (eMethods).

### Statistical analyses

#### Generalizability of pseudotime and subtypes

We first tested the generalizability of the molecular AD dementia pseudotime by projecting it onto 223 additional ROSMAP participants with matched omic data who were not included in the initial analysis, and separately onto 118 participants from the Mount Sinai Brain Bank (MSBB; synapse ID:syn3159438) [57, 58]. Generalizability of the AD subtypes was assessed only in the 223 ROSMAP participants since the MSBB sample size (N = 118) was insufficient to reliably project subtypes (eMethods). To test generalizability, we cross-validated the subtypes from the discovery-sample (n = 844) with subtypes independently generated in the 223 ROSMAP participants as described in the eMethods. Concordance was quantified using normalized mutual information (nMI), a measure of mutual dependence between the two classification configurations, assessed via 10,000-label permutation tests. Because the pseudotime and subtype solutions have previously been replicated and generalized to peripheral blood omics in an independent living cohort(ADNI [34]), we did not re-evaluate translational or clinical validity here.

#### Annotation of molecular AD subtypes

We annotated top (FDR *p* < 0.05) molecular features characterizing the three previously reported subtypes [34]. For each subtype, we described the proportion of top 25 omic features.

#### Associations with neuropathology

As pseudotime and the subtypes are built from AD dementia as the target, we compared frequency of pathologies across subtypes to ensure that our molecular subtypes were not simply capturing different pathological features. Pathologies were grouped as:(i) AD and CAA; (ii) non-AD neurodegenerative (neocortical Lewy bodies, TPD-43, hippocampal sclerosis); (iii) total infarcts, (iv) cerebral vessel pathology: arteriosclerosis, atherosclerosis. Chi-squared tests were used to determine whether observed frequencies differed by subtype.

#### Associations with brain morphometry

We examined voxel-wise deformation using general linear-models using FSL PALM [59, 60] adjusting for demographics, postmortem interval, and scanner(accounting for both mean and variance differences across scanners). We then contrasted each group pair. P-values were computed from 500 permutations using tail approximation [61]. The threshold-free cluster enhancement approach was used to define clusters of significance. Associations were considered statistically significant at p ≤ 0.05, family-wise error rate corrected.

#### Associations with polygenic risk-scores

We assessed the overall correlation of 802 polygenic risk-scores with pseudotime using Elastic Net regression with 10-fold cross-validation and evaluated their collective predictive capacity in distinguishing amongst the subtypes using Elastic Net classifiers with 10-fold cross-validation, with classifier performance measured by area under the receiver operating curve(AUC). Because all PGS were entered into a single cross-validated Elastic Net model, classical multiple correction was not required.

#### Associations with psychological traits

Finally, we assessed relationships between psychological traits and pseudotime in four separate linear regression models adjusted for age at baseline, sex, education, and time to death. We further examined trait associations with subtype assignment using multinomial logistic regression, adjusting first for the same covariates, and in additional models adjusting further for the listed neuropathologic indices. All trait measures were standardized to enable direct comparison across traits and facilitate comparability.

## Results

A total of 822 participants had multi-omic data. Of those, 761 had neuroticism scores, 818 had CES-D data, and 306 participants had measures on loneliness and purpose which were only available in MAP. Mean age at baseline was just over 80 years and mean age at death was close to 90 years. Over 60% were female, and most participants completed college. Over a quarter carried the APOEε4 allele, with more than a third of individuals in Subtype AD2 carrying this variant (χ^2^ (3819) = 22.0, p < 0.001; Table 1).

**Table 1:** Characteristics of the whole sample and stratified by controls and AD subtypes.

	Mean (SD) or % (*n*)				
Characteristics	Whole sample	NCI	Subtype AD1	Subtype AD2	Subtype AD3
N	822	274	189	197	162
Age at baseline, mean years, (SD)	82.3 (6.7)	79.2 (6.9)	81.9 (6.3)	82.2 (6.8)	82.9 (6.9)
Age at death, mean years (SD)	88.4 (6.6)	86.2 (6.5)	89.3 (6.4)	89.1 (6.6)	90.4 (6.1)
Female, n (%)	532 (64)	171 (32)	130 (24)	122 (22)	109 (20)
Educational attainment, mean years (SD)	16.3 (3.5)	16.3 (3.6)	16.4 (3.6)	16.2(3.5)	16.3 (3.2)
Neuroticism, baseline, (SD)	16.8 (6.6)	15.8 (6.7)	17.8 (6.6)	16.7 (6.4)	17.5 (6.3)
Depressive symptoms, mean score (SD)	1.28 (1.65)	1.13 (1.5)	1.37 (1.8)	1.35 (1.6)	1.37 (1.8)
Loneliness, baseline (SD)	2.4 (0.6)	2.2 (0.6)	2.4 (0.6)	2.3 (0.5)	2.5 (0.7)
Purpose in life, baseline (SD)	3.6 (0.5)	3.6 (0.5)	3.5 (0.4)	3.5 (0.4)	3.5 (0.4)
Pseudotime, mean (SD)	0.4 (0.21)	0.23 (0.13)	0.38 (0.13)	0.45 (0.09)	0.68 (0.18)
Global cognition, baseline, mean (SD)	−0.23 (0.7)	0.17 (0.4)	−0.40 (0.6)	−0.45 (0.7)	−0.43 (0.9)
Global cognition, last valid, mean (SD)	−0.96 (1.2)	0.08 (0.4)	−1.45 (1.1)	−1.50 (1.1)	−1.50 (1.1)
Mild cognitive impairment proximate to death, n (%)	197 (23)	0	63 (31)	78 (39)	56 (28)
AD dementia proximate to death, n (%)	351 (42)	0	126 (35)	119 (33)	106 (30)
APOEe4, any e4, n (%)	214 (26)	44 (16)	63 (34)	57 (29)	50 (31)
Neuropathology					
Pathologic AD (NIA-AA), n (%)	517 (62.9)	118 (43.1)	141 (74.6)	138 (70.1)	120 (74.1)
Neocortical Lewy bodies, n (%)	84 (10.2)	13 (4.7)	18 (9.5)	28 (14.2)	25 (15.4)
TDP-43, n (%)	238 (30.4)	43 (16.5)	64 (36.4)	72 (38.3)	59 (37.3)
Hippocampal sclerosis, n (%)	60 (7.4)	6 (2.2)	17 (9.0)	25 (12.8)	12 (7.5)
Gross chronic infarcts, n (%)	294 (35.8)	61 (22.3)	83 (43.9)	77 (39.1)	73 (45.1)
Micro chronic infarcts, n (%)	225 (27.4)	63 (23.0)	60 (31.7)	56 (28.4)	46 (28.4)
Arteriosclerosis, moderate to severe, n (%)	317 (38.9)	78 (28.7)	74 (39.4)	99 (50.8)	66 (41.3)
Atherosclerosis, moderate to severe, n (%)	346 (42.2)	86 (31.7)	84 (44.4)	98 (49.7)	78 (48.1)
Cerebral amyloid angiopathy, moderate, n (%)	281 (35.1)	75 (28.2)	69 (37.1)	73 (38.4)	64 (40.3)

### Portability of pseudotime and subtypes

In the replication samples, average age at death was 90.7 years (SD = 7.1) in ROSMAP and 79.2 years (SD = 7.1) in MSBB. 71%(ROSMAP) and 63%(MSBB) were female.

**Fig. 1:**
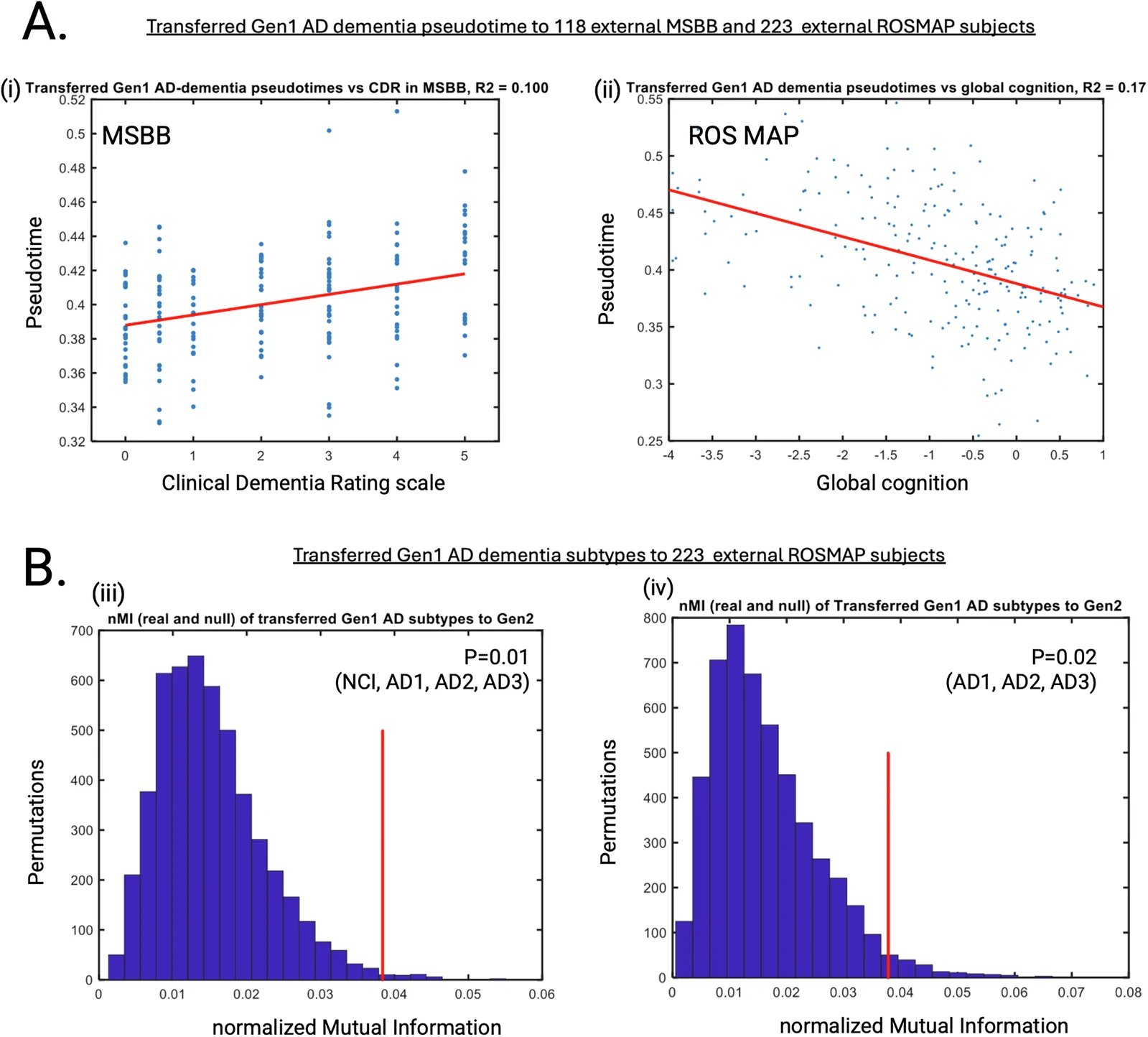
Replication of pesudotime and AD subtypes. **A** Projection of brain molecular multi-omic AD Pseudotime to independent samples. The figures in the top panel illustrate the association between the projected multi-omic brain molecular Pseudotime on the y-axis and the (i) Clinical Dementia Rating scale, where higher scores denote more severity in the Mount Sinai Brain Bank (MSBB) and (ii) global cognition where lower scores denote more severity in independent ROSMAP participants, on the x-axis. **B** Projection of brain molecular multi-omic AD subtypes to independent samples. The figure shows the null distribution of the nMI values generated from 10,000 permutation tests (the purple histogram) assessing the similarity between subtype classification transferred from the discovery to the independent ROSMAP samples. The red vertical line denotes the observed nMI between the actual and the projected subtypes. Since the observed value (red line) lies far to the right of the distribution, it indicates significant greater correspondence between the subtype configurations than expected by chance both when we (iii) include the pre-defined background group, and when we (iv) exclude it, thus assessing only projected subtypes.

The projected molecular pseudotime significantly predicted clinical status (MSBB) and global cognition (ROSMAP)(all p < 0.001), indicating that our multi-omic molecular AD pseudotime can be robustly extended to independent samples (Fig. 1A).

Subtype projection in 223 independent ROSMAP participants revealed significant concordance (nMI p = 0.02). These results indicate strong mutual dependence and robust generalizability of the subtypes across datasets (Fig. 1B).

### Annotation of molecular AD subtypes

The AD subtypes were derived after we decomposed the multi-omic brain molecular pseudotime, which top molecular features included dysregulation of the HOXC9 gene at the RNA level, altered states of lithocholic acid at the metabolomic level, and altered expression of tau 77G7 at the proteomic level [34].

**Fig. 2:**
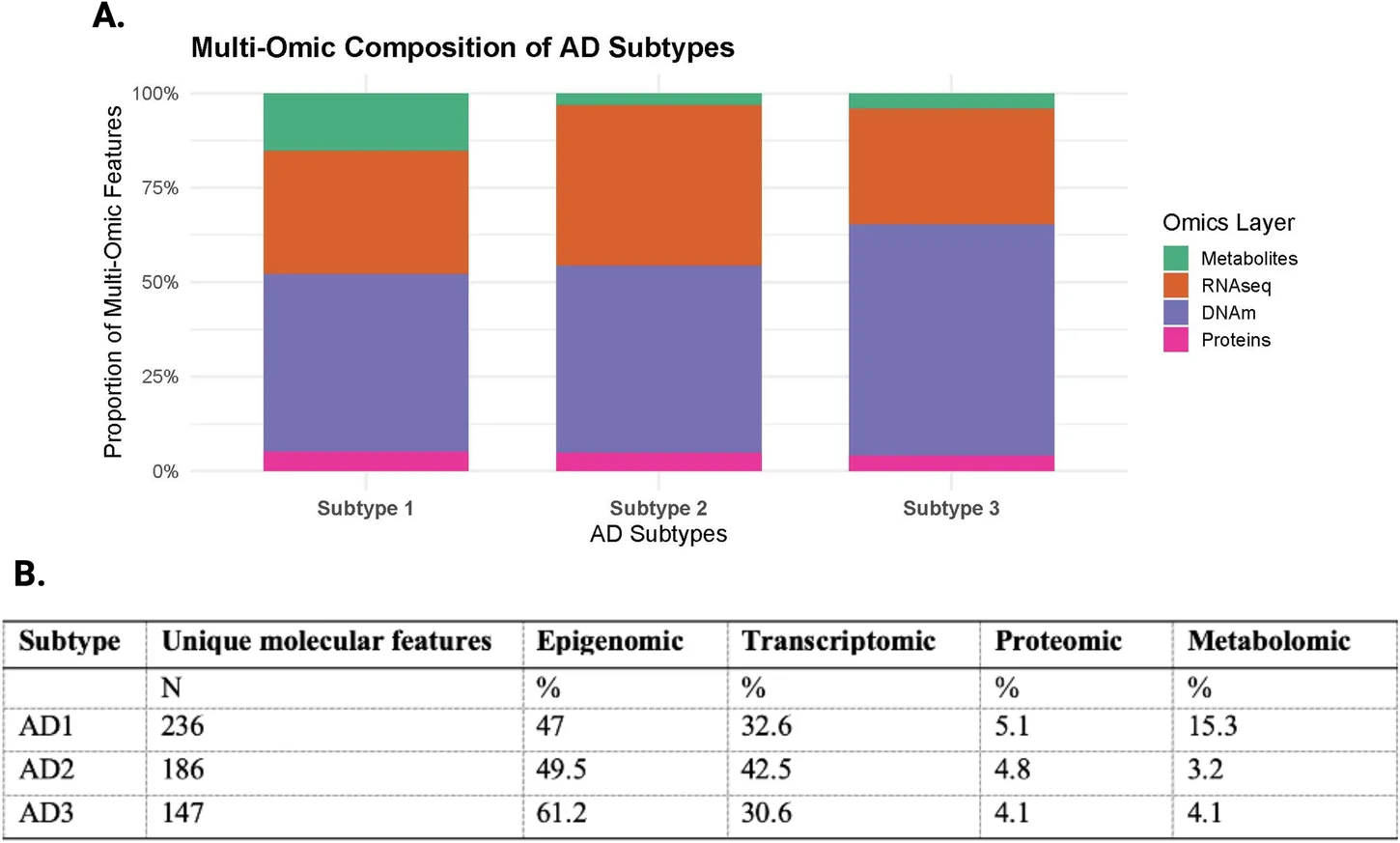
Distribution and proportion of altered omic molecular alterations across AD subtypes. **A** Distribution of altered molecular features (epigenomic, transcriptomic, proteomic, and metabolomic) across the three identified AD subtypes. Each colored bar adds up to 100% for that particular AD subtype. Therefore, while altered epigenomic features were the most common omic modality in all subtypes, relative to each other, AD1 was characterized by metabolomic alterations, AD2 by transcriptomic alterations, and AD3 by epigenomic alterations. Only markers significantly associated at FDR *p* < 0.05 were considered in this study. **B** Proportions of omic alterations within each AD subtype. Values in each row sum to 100%, reflecting relative contribution of each omic layer to the subtype’s total unique alterations.

We first identified omic features that differentiated AD subtypes from NCI. Since some omic alterations were shared across subtypes, we restricted our focus to omic features that were significantly altered in one subtype but not the others. These subtype-defining features reflect molecular changes that are uniquely and differentially enriched in each subtype. While most distinctive alterations of all subtypes relative to NCI happened at the epigenetic and transcriptomic levels (Fig. 2A), each subtype was characterized by a unique set of non-overlapping differentiating features (see S.Table 2). Specifically, AD1 was characterized by 236 uniquely differentiated features, AD2 by 186, and Subtype 3 by 147 unique features. Thus, AD1 was characterized by 21% more molecular alterations than AD2 and 37.8% more so than AD3, suggestive of more rapid progression of the disease than the other two subtypes. Relative to each other, AD1 showed the highest proportion of metabolite alterations (~15%), AD2 was mostly enriched for transcriptomic changes (~43%), and AD3 was characterized by extensive epigenomic dysregulation (>60%) (Fig. 2B). These results provided insight into the underlying biology of each subtype. AD1 may reflect greater downstream disruption in cellular metabolism and energy-related biochemical activity. AD2 may indicate that more genes are being expressed or regulated differently. AD3 dominated by epigenetic changes suggests gene-regulatory mechanisms for this subtype may be more upstream and thus disrupted earlier or stronger in this subtype. To this extent, AD1 might be a Metabolomic-Dominant Subtype; AD2 a Transcriptomic-Dominant Subtype; and AD3 Epigenetic-Dominant Subtype, although we note that these descriptions are provisional and that future studies are needed to identify causal mechanisms and clearly define such subtypes.

**Fig. 3:**
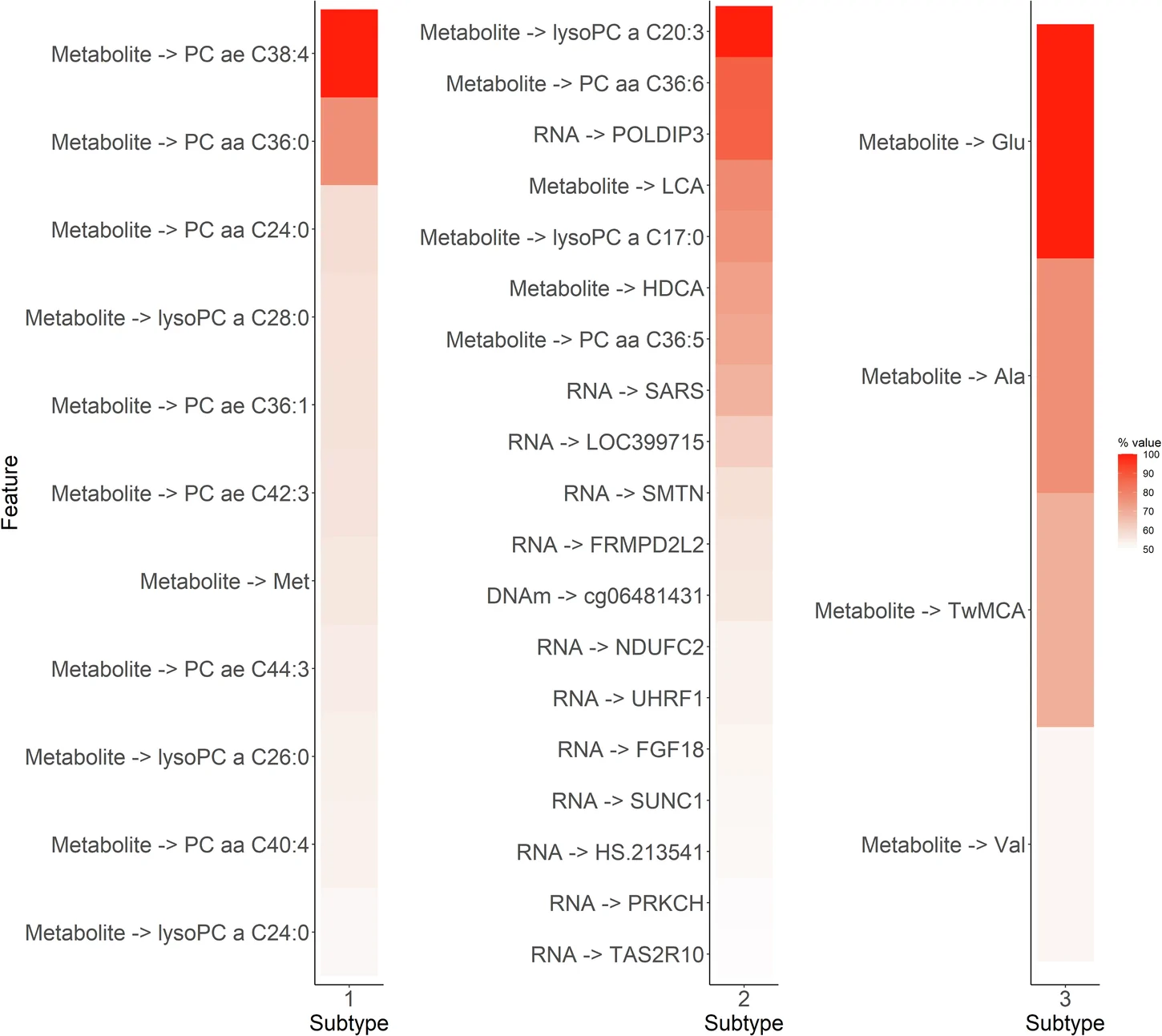
Top omic features per AD subtype. Top molecular omics features of AD molecular subtypes in the DLPFC of the postmortem human brain. Top influential metabolomic, transcriptomic, epigenomic and proteomic markers per AD subtype (F-values in percentages, only F-values > 50% of omic signals are shown). In all, while epigenomic features were the most frequent data modality across subtypes, the strongest features as determined by the F-value, consistently were metabolites.

Of the omic features that distinguished the subtypes from NCI, top distinguishing omic features (highest f-values) were metabolites (F-value > 50%; Fig. 3). The strongest defining features (F-value > 90%) differentiating the subtypes from NCI were three phospholipids (phosphatidylcholine acyl-alkyl (PCae)C38:4 for AD1; lysophosphatidylcholine (lysoPC)acyl C20:3 and PC acyl-acyl (aa)C36:6 for AD2;), and a major excitatory neurotransmitter (glutamate) for AD3.

Across all omic features, AD2 consistently exhibited higher F-values (s.Fig. 1), indicating more pronounced molecular divergence from NCI than AD1 and AD3 (sTables 3–4).

### Frequency of AD/ADRD pathologies across AD subtypes

The NCI group had fewer pathologies, as expected (median = 2), whereas AD subtypes had a median of 3. Over half of NCI participants had 1–2 pathologies, while over two thirds of participants across all subtypes had ≥3, which is expected since mixed pathologies commonly drive AD dementia (sTable 5).

>70% of individuals across all subtypes had pathologic AD and CAA (χ^2^(2535) = 1.96, *p* = 0.38), and almost half had non-AD neurodegenerative neuropathology (χ^2^(2517) = 4.09, *p* = 0.66). There were no differences in the distribution of infarcts ((χ^2^(2548) = 4.01,*p* = 0.14) or in frequency of cerebral vessel neuropathology (χ^2^(4543) = 2.89,*p* = 0.24) (sFig. 2).Therefore, our molecular subtypes are not simply capturing different neuropathological features.

### Brain morphology across AD subtypes

Amongst 278 participants (NCI = 91, AD subtypes: 1 n = 74; 2 n = 57, 3 n = 56) with postmortem MRI, all AD subtypes exhibited greater cortical atrophy, relative to NCI (sFig. 3). Subtype AD1 exhibited the most extensive atrophy, as observed by smaller volumes in the medial temporal lobe and across temporal, frontal, and parietal regions. Subtype AD2 exhibited temporal lobe atrophy that extended to the temporal pole. Subtype AD3 exhibited the least atrophy, which was largely localized to the temporal lobe and insular cortex. Both Subtypes AD1 and AD2 also had extensive ventricular enlargement, which indicated central brain atrophy, while Subtype AD3 had minimal enlargement of the temporal horn. However, inter-subtype contrasts were statistically non-significant likely due to limited sample size.

### Prediction of polygenic risk scores for pseudotime and subtypes

When analyzing the full cohort including background and subtypes together, we observed a significant overall association between polygenic risk scores and pseudotime (r = 0.19, p = 3 × 10-8), suggesting that genetic variation is meaningfully associated with disease progression across the entire spectrum (sFig. 4A). However, when restricting the analysis to AD subtypes only, polygenic risk scores were no longer able to predict pseudotime, suggesting that PRS are better associated with molecular disease progression, indicative of general disease liability, but not with the subtypes.

Similarly, when we tested these polygenic risk scores for subtype classification, scores moderately differentiated AD subtypes from the background group (AUC ~ 0.70), but they did not effectively distinguish among the individual AD subtypes themselves (AUC < 0.60). These results suggest that genetic risk scores are better at identifying participants from the background group than at discriminating between individual AD subtypes (sFig. 4B-C).

### Associations with psychological traits

Pseudotime increased progressively across subtypes with higher pseudotime indicating closer proximity to AD dementia (Table 1).

Higher neuroticism (est.=0.019, SE = 0.008 *p* = 0.01) and loneliness (est.=0.025, SE = 0.012, *p* = 0.04) were associated with higher pseudotime, explaining 4% and 2% of variance. CES-D and purpose were not associated.

**Fig. 4:**
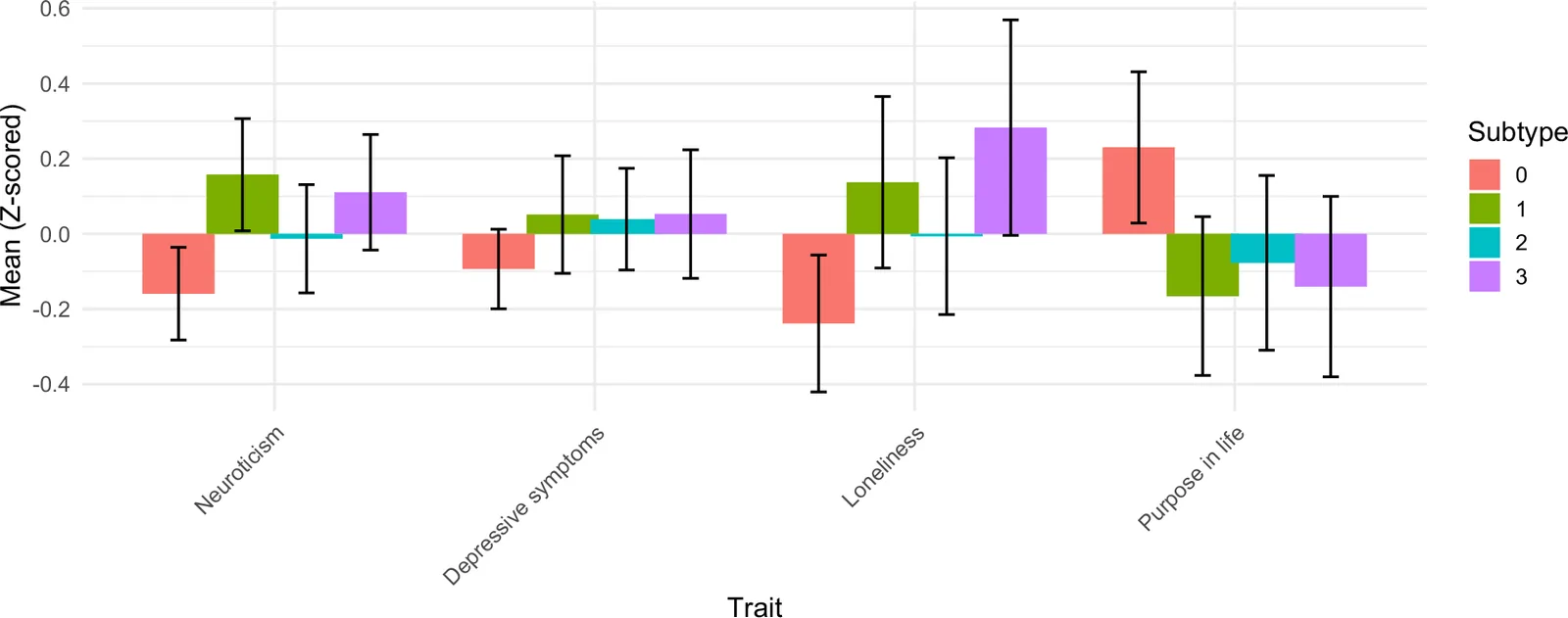
Distribution of the mean level of four psychological traits across the AD subtypes. Bar plots displaying distribution of the z-scored mean level of each psychological trait (neuroticism, depressive symptoms, loneliness, and purpose in life) across the no cognitive impairment group (background = 0) and the AD subtypes (1 = AD1, 2 = AD2, 3 = AD3). As indicated by the orange bars, the background had lower mean levels of neuroticism, depressive symptoms, loneliness, and higher mean levels in purpose in life. The confidence intervals show that while inter-subtypes means are not different from each other, neuroticism and loneliness differentiated NCI from some but not all AD subtypes. This is a descriptive plot, and no adjustments were made.

Relative to background, all AD subtypes had higher mean levels of neuroticism, depressive symptoms, and loneliness, and lower mean levels on purpose though confidence intervals overlapped for the most part (Fig. 4).

**Fig. 5:**
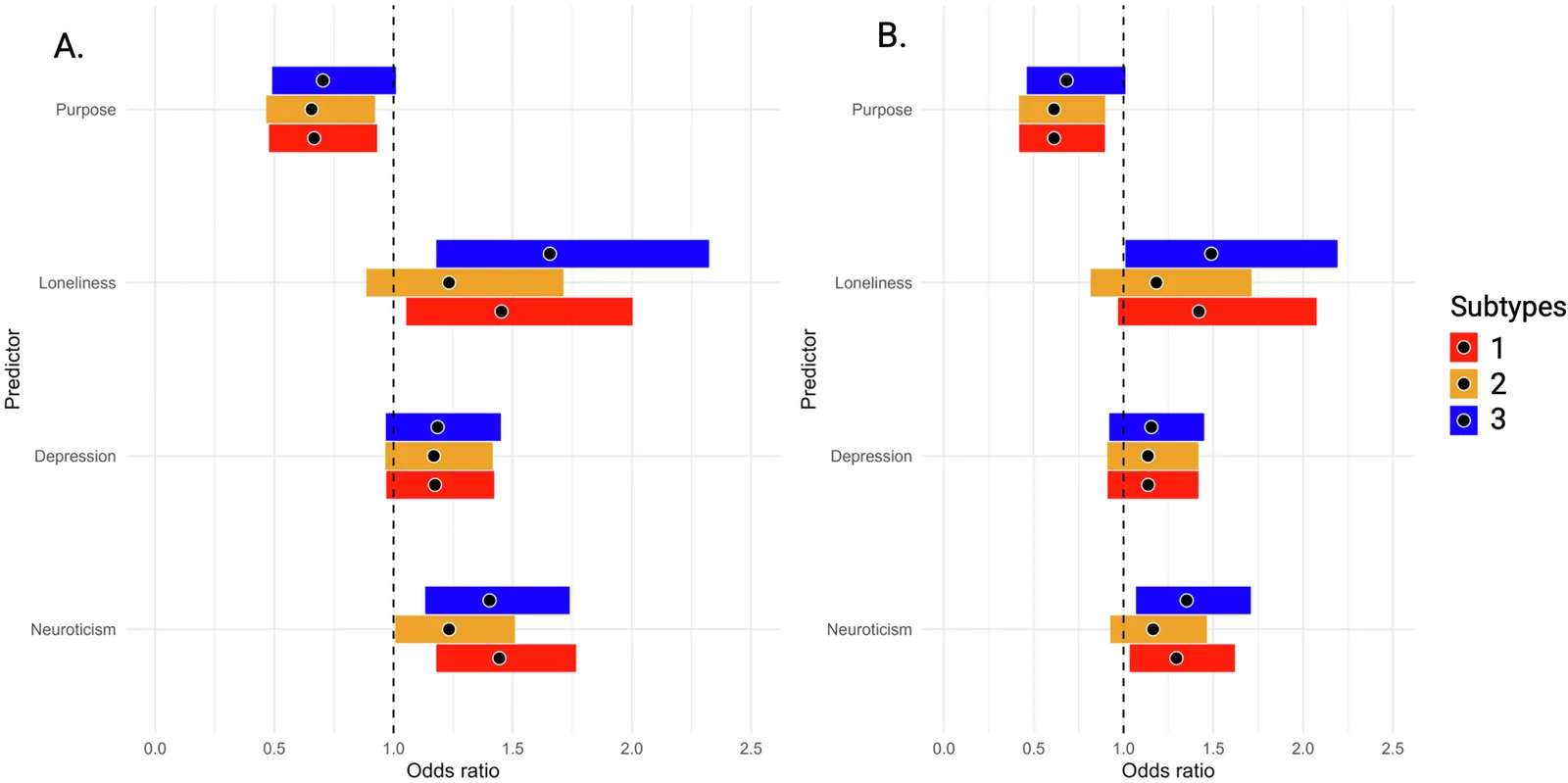
Forest plot showing the odds ratios of belonging to AD subtypes before and after adjusting for nine common ADRD neuropathologic indices. **A** Forest plots displaying the odds of belonging to AD subtypes 1 (red bar), 2 (yellow bar), or 3 (blue bar), relative to the no cognitive impairment group adjusting for age, sex, education, and time to death. **B** Forest plots further adjusting for 9 ADRD neuropathologic indices. Each row shows the odds (black dot) and confidence interval (shaded rectangles) of the psychological traits (y-axis) on belonging to AD1 (red), AD2 (orange), or AD3 (blue) relative to being in the no cognitive impairment (NCI) group. Odds on the right from 1 represent higher odds of belonging to AD subtypes relative to NCI, while odds from the left of 1 represent lower odds of belonging to these subtypes.

Higher neuroticism increased odds of belonging to each AD subtype relative to NCI: (AD1:OR = 1.44, 95%CI = 1.18–1.98, p < 0.001; AD2:OR = 1.23, 95%CI = 1.01–1.51, p = 0.042; AD3:OR = 1.40, 95%CI = 1.13–1.74, p = 0.002). Greater loneliness increased odds for AD1 (OR = 1.43, 95%CI = 1.05–2.00, p = 0.02) and AD3 (OR1.66, 95%CI = 1.18–2.32, p = 0.004), but not for AD2. By contrast, higher purpose in life was associated with lower odds for AD1 (OR = 0.67, 95%CI = 0.48,0.93, p = 0.02) and AD2 (OR = 0.66, 95%CI = 0.47–0.92, p = 0.02). Depressive symptoms showed no associations (Fig. 5A).

In models adjusting further for nine ADRD neuropathologic indices, neuroticism remained significantly associated with AD1 (OR = 1.30, 95%CI = 1.04–1.62, p = 0.02) and AD3 (OR = 1.35, 95%CI = 1.07–1.71, p = 0.01). Loneliness remained associated with AD3 (OR = 1.49, 95%CI = 1.01–2.19,p = 0.04), while purpose remained inversely associated with AD1 (OR = 0.61, 95%CI = 0.42–0.90, p = 0.01) and AD2 (OR = 0.61, 95%CI = 0.41–0.90,p = 0.01). Results indicate that these traits are associated with these molecular subtypes of AD independent of neuropathology (Fig. 5B).

After adjusting for multiple comparisons across the four traits, the direction of associations remained the same though only associations of Neuroticism with AD1 and associations of purpose with AD1 and AD2 remained significant.

## Discussion

In this exploratory study, we build on prior work in 822 older adults who subsequently died and had brain autopsy, providing brain multi-omic molecular pseudotime, and three distinct molecular subtypes representing pathways from NCI to AD dementia. We characterized the subtypes across neuropathological burden, brain atrophy, genetic susceptibility, and psychological risk factors. Neuropathologic burden, brain morphology, and polygenic risk differentiated the subtypes from NCI but they did not differentiate amongst subtypes. By contrast, neuroticism, loneliness, and purpose in life showed differential associations with the AD subtypes in that some but not all traits were associated with the subtypes, even after we adjusted for neuropathology, which was evenly distributed across the subtypes. Results suggest that psychological risk factors of AD dementia may align with distinct molecular signatures of AD dementia beyond the presence of pathology, consistent with a two-stage model in which upstream genetic risk influences overall disease liability, while psychological predispositions relate more specifically to subtype differentiation across the heterogeneous AD spectrum not explained by neuropathologic burden or brain atrophy patterns. Psychological traits may thus be associated with AD dementia via multi-omic molecular pathways.

Previous studies have largely examined single-omic associations to explore the molecular biology underlying these traits. Transcriptome- and proteome- wide association studies have identified genes and proteins associated with neuroticism [62], depressive symptoms [63, 64], and loneliness [64, 65]. DNA methylation signatures, and separately metabolomic signatures of depressive symptoms have also been reported with enrichment analyses highlighting metabolites involved in inflammation, glycerides and phospholipids [66], as well as methylation sides implicated in axon guidance as a disrupted pathway [67]. Proteomic signatures of loneliness have also been identified with evidence suggesting causal associations between loneliness and proteomic expression [68, 69]. Moreover, loneliness [70] and depression [71] have each been associated with accelerated epigenetic age, whereas purpose in life has been associated with reduced epigenetic age [72]. In addition, purpose in life has been associated with improved mitochondrial function [73] and with lower systemic inflammation [74]. Plasma omics is also being used to explore proteins associated with incident depression [75–77] and anxiety [76]. Similar work is being doing using plasma metabolomics [67, 78]. However, our study sought to move a step forward by exploring whether these risk factors might be associated with one of more molecular pathways associated with AD dementia.

We previously showed that neuroticism is associated with 18 cortical transcriptomic co-expressed modules, three of which partially mediated the association of higher neuroticism on faster cognitive decline [33]. We previously also identified two cortical proteins, 40S ribosomal protein S3 and BCKDHB, that were associated with both neuroticism and cognitive decline independent of common AD/ADRD pathologic indices [79]. These two proteins strongly mediated the association of higher neuroticism on cognitive decline independent of common AD/ADRD pathologic indices, and further explained 25% of the variance in this association. Together, these studies provide evidence that neuroticism has strong and widespread effects on the transcriptome, and on specific cortical proteins in the aged prefrontal cortex, both of which affect downstream AD-related outcomes. Similarly, while in prior work we identified cortical proteins associated with depressive symptoms [63], we further identified a cortical protein, IGFBP-5, that was associated with both depressive symptoms and cognition, and further explained 10% of their association [80]. We also previously identified four microRNAs associated with depressive symptoms, of which miR-484 targets were enriched in a co-expression module involved in synaptic function and plasticity and were associated with higher risk of AD dementia [81]. However, the shared molecular mechanisms between these traits and AD/ADRD remain still largely unexplored.

We extend prior work by characterizing multi-omic, molecularly defined subtypes of AD dementia, and linking them to neuropathology, brain atrophy, genetic susceptibility, and psychological risk factors. Our data suggest a two-stage model of i) overall disease liability, and ii) downstream subtype differentiation. Specifically, polygenic risk was associated with molecular disease progression (pseudotime), but it did not differentiate amongst the subtypes, whereas phenotypic trait expression did. We interpret this as evidence that genetic variation reflects overall AD susceptibility, while the subtypes capture downstream risk differentiation. In this framework, individuals with similar genetic risk and similar molecular disease progression (stage 1), may nevertheless have distinct molecular signatures that are subjected to different behavioral risk factors (stage 2). Indeed, we found that psychological risk factors were differentially associated with subtype-specific molecular signatures of AD dementia, even after adjusting for common AD/ADRD pathologies, indicating that these traits may align with molecular pathways of AD dementia beyond pathology. Furthermore, because the pathologies did not differentiate amongst the subtypes, our results further indicate that the multi-omic subtypes capture variance beyond that explained by ADRD pathologies. To our knowledge, this is the first study that has leveraged multi-omics data to examine how psychological traits relate to distinct molecular configurations of AD dementia. This approach allowed us to identify differential associations between four psychological traits and three distinct molecular AD subtypes. Much work remains to expand our findings in better-powered studies, with additional and larger multi-layered omic subtypes to elucidate potential molecular pathways linking these risk factors to AD dementia.

Our findings suggest that the various AD molecular mechanisms that are subserving cognition are also related to psychological traits. Consistent with prior plasma-based studies [82, 83], metabolomic alterations were present across all subtypes, and top features in AD1 and AD2 were phosphatidylcholines(PCs), key components of cell membranes with roles in lipid structure and signaling. Reductions in several PC species, including ae c38:4 and PC aa C36:6 are associated with clinical AD [84–86], and lysoPCs, are proinflammatory lipids, substantially highly concentrated in AD [87]. Glutamate is a major excitatory neurotransmitter in the central nervous system, which overactivation has been implicated in neuronal damage and linked to AD-related synaptic vulnerability [88]. These mechanisms are postulations, and they do not imply causal pathways; however, they highlight the molecular domains that most strongly differentiate the subtypes from NCI in the dataset. Further studies with additional omic layers will be needed to better characterize these multi-omic AD subtypes and their potential relevance in linking psychological traits to clinical AD.

The current study was limited to the data available. Psychological assessment was self-reported, and biomarkers were collected postmortem. The brief measures reflecting neuroticism and depressive symptoms, may have underestimated associations, though we previously established their validity and replicability [17, 89]. Measures of loneliness and purpose were only available in MAP participants, reducing statistical power. We did not utilize in-vivo indicators such as PET imaging, or plasma markers; indeed, pseudotime was developed in part to address this limitation by transforming cross-sectional post-mortem multi-omic data into an ordered continuum reflecting molecular progression time [90, 91]. Importantly, we had gold-standard post-mortem data that reflected accumulated pathologic burden and multi-omic molecular landscape across the DLPFC, something that in-vivo biomarkers rarely capture. Because many biological processes relevant to AD evolve over decades, postmortem molecular profiles are understood to reflect chronic, long-standing biological states. Thus, even with an ~10-year time gap between psychological assessment and brain tissue collection, evaluating stable psychological traits in relation to postmortem brain measures provides meaningful insight into how these traits relate to AD-related molecular signatures. Finally, all omics came from one brain region and the number of epigenomic and transcriptomic features were disproportionally higher than those for metabolites or proteins [34], which might have overestimated the noted proportions of epigenomic features within subtypes. However, the DLPFC is a known region for its associations with learning, stress response and emotion regulation, and the continuous collection of omics data within our cohorts will allow for further multilevel characterization of complex traits and replication of results. Our replicated AD dementia pseudotime and subtypes in independent samples testify to their generalizability. Our novel multi-omic approach provides insights into the molecular basis of psychological AD/ADRD risk factors. Other approaches have been used to identify molecular subtypes of AD; however, they have been agnostic to any clinical trait [92–95]. Finally, while mechanistic validation will be essential in future studies, this approach offers a framework for identifying molecular signatures and generating testable hypotheses about how long-standing psychological risk factors intersect with AD biology.

## Supplementary information


S. Table 1
Supplementary Material
eMethods


## Data Availability

The data are available via the Rush Alzheimer’s Disease Center Research Resource Sharing Hub. Qualified applicants should complete an application including study premises and a brief description of the research plan. Brain multi-omic data are available in the AMP-AD knowledge portal (www.synapse.org) using the following Synapse IDs:syn3157275 (epigenomic data), syn3800853 (transcriptomic), syn10468856 (proteomic), and syn10235595 and syn10235594 (metabolomic).
