# Supplementary material for: Associations of stable psychological traits with multi-omic subtypes of Alzheimer’s dementia
Source: Transl Psychiatry. 2026 Jun 21;16:443. doi: 10.1038/s41398-026-04195-z (PMC13530231; doi:10.1038/s41398-026-04195-z)
Supplement: Supplementary file 1 [file 41398_2026_4195_MOESM2_ESM.docx]

**Supplementary Material**

**Tables**

**S.Table 2.** Full list of omic signals contributing to the three AD subtypes at FDR *p* <0.05.

| **AD subtype 1** | **AD subtype 2** | **AD subtype 3** |
| --- | --- | --- |
| Metabolite -> PC ae C38:4; Metabolite -> PC aa C36:0; Metabolite -> PC aa C24:0; Metabolite -> lysoPC a C28:0; Metabolite -> PC ae C36:1; Metabolite -> PC ae C42:3; Metabolite -> Met; Metabolite -> PC ae C44:3; Metabolite -> lysoPC a C26:0; Metabolite -> PC aa C40:4; Metabolite -> lysoPC a C24:0; Metabolite -> PC aa C42:5; Metabolite -> PC ae C40:4; Metabolite -> PC ae C40:5; Metabolite -> Cit; Metabolite -> PC aa C26:0; Metabolite -> PC ae C36:0; Metabolite -> PC ae C38:3; Metabolite -> C14:2-OH; Metabolite -> C14:1-OH; Metabolite -> PC aa C42:4; Metabolite -> His; Metabolite -> PC ae C44:6; Metabolite -> PC ae C30:0; Metabolite -> PC ae C38:2; Metabolite -> C10; Metabolite -> PC ae C34:1; Metabolite -> PC aa C30:0; Metabolite -> C14:2; Metabolite -> lysoPC a C26:1; Metabolite -> Orn; Metabolite -> SDMA; Metabolite -> PC aa C40:3; Metabolite -> lysoPC a C28:1; Metabolite -> PC ae C36:4; Metabolite -> PC ae C36:2 (RNA -> FXYD7; RNA -> S100PBP; RNA -> LOC642450; RNA -> TFPT; RNA -> TRIML2; RNA -> C21ORF34; RNA -> GRK4; RNA -> WDR19; RNA -> PLAC8L1; RNA -> NSUN5C; RNA -> SNRP70; RNA -> MAST1; RNA -> TMEM50A; RNA -> FLJ39061; RNA -> MYADM; RNA -> FNBP4; RNA -> HS.391327; RNA -> MESP1; RNA -> HS.538763; RNA -> LOC651125; RNA -> HSPC111; RNA -> IDUA; RNA -> C21ORF114; RNA -> MGC27345; RNA -> LOC650155; RNA -> TNFRSF25; RNA -> LOC388237; RNA -> HS.458154; RNA -> HS.532239; RNA -> NPIP; RNA -> LST-3TM12; RNA -> PDE5A; RNA -> KRT13; RNA -> LAMA5; RNA -> LAMA3; RNA -> LOC731042; RNA -> ELL3; RNA -> NXF1; RNA -> TRAPPC6A; RNA -> PSMD5; RNA -> HS.576530; RNA -> CAPNS1; RNA -> CHKB; RNA -> HS.581313; RNA -> ABL2; RNA -> TAZ; RNA -> GOLGA8G; RNA -> PBX4; RNA -> SIL1; RNA -> HIST1H2BO; RNA -> MYOC; RNA -> HS.407028; RNA -> HSF4; RNA -> LOC647371; RNA -> ZNF649; RNA -> GTPBP8; RNA -> SCPEP1; RNA -> HS.105791; RNA -> LOC650887; RNA -> LOC652168; RNA -> POLR3F; RNA -> SETD2; RNA -> HS.570762; RNA -> ACADVL; RNA -> LOC644143; RNA -> TPPP3; RNA -> LOC642980; RNA -> TIMM17B; RNA -> TENC1; RNA -> C14ORF148; RNA -> MBTPS1; RNA -> PTPRE; RNA -> CCDC43; RNA -> FLJ41481; RNA -> GTF2IRD2B; RNA -> HS.559929; RNA -> HS.314177 (DNAm -> cg11278506; DNAm -> cg10104252; DNAm -> cg14074486; DNAm -> cg22176017; DNAm -> cg11995069; DNAm -> cg26709695; DNAm -> cg15850287; DNAm -> cg16668548; DNAm -> cg25664220; DNAm -> cg08277100; DNAm -> cg19360330; DNAm -> cg20911707; DNAm -> cg13027384; DNAm -> cg15077558; DNAm -> cg18574476; DNAm -> cg07285896; DNAm -> cg17204018; DNAm -> cg23517743; DNAm -> cg05005586; DNAm -> cg14664242; DNAm -> cg00678938; DNAm -> cg08005122; DNAm -> cg25061682; DNAm -> cg04134031; DNAm -> cg23894539; DNAm -> cg05809481; DNAm -> cg14270725; DNAm -> cg16652332; DNAm -> cg00844791; DNAm -> cg13638257; DNAm -> cg06002947; DNAm -> cg01809217; DNAm -> cg23002008; DNAm -> cg12833207; DNAm -> cg06565615; DNAm -> cg08030134; DNAm -> cg19131647; DNAm -> cg17696847; DNAm -> cg08787039; DNAm -> cg00167958; DNAm -> cg02186298; DNAm -> cg06837765; DNAm -> cg22425711; DNAm -> cg26875637; DNAm -> cg15964611; DNAm -> cg05682952; DNAm -> cg21951594; DNAm -> cg20801456; DNAm -> cg01233948; DNAm -> cg22222413; DNAm -> cg17623882; DNAm -> cg19045313; DNAm -> cg00719108; DNAm -> cg00517796; DNAm -> cg07537581; DNAm -> cg18426156; DNAm -> cg21202355; DNAm -> cg22839075; DNAm -> cg17961892; DNAm -> cg14019695; DNAm -> cg09126038; DNAm -> cg06529685; DNAm -> cg24080119; DNAm -> cg17847344; DNAm -> cg03978041; DNAm -> cg06514855; DNAm -> cg26383138; DNAm -> cg16877615; DNAm -> cg13126315; DNAm -> cg00730653; DNAm -> cg25623271; DNAm -> cg07366506; DNAm -> cg01173186; DNAm -> cg02225716; DNAm -> cg26404886; DNAm -> cg09002677; DNAm -> cg23601614; DNAm -> cg06684407; DNAm -> cg20586124; DNAm -> cg27102141; DNAm -> cg10805850; DNAm -> cg27364588; DNAm -> cg09602803; DNAm -> cg02319733; DNAm -> cg24702091; DNAm -> cg09914513; DNAm -> cg19727817; DNAm -> cg02498382; DNAm -> cg21624342; DNAm -> cg14866339; DNAm -> cg15866213; DNAm -> cg10736454; DNAm -> cg02661886; DNAm -> cg15646626; DNAm -> cg00081729; DNAm -> cg10333808; DNAm -> cg03070187; DNAm -> cg19783404; DNAm -> cg01382100; DNAm -> cg18504937; DNAm -> cg10427793; DNAm -> cg14366742; DNAm -> cg17437621; DNAm -> cg15336438; DNAm -> cg00047185; DNAm -> cg07003632; DNAm -> cg11942329; DNAm -> cg27357865; DNAm -> cg22429418; DNAm -> cg27085265; DNAm -> cg19701084; (Protein -> NDUFA10_1; Protein -> HSPA8_1; Protein -> DOCK2; Protein -> NDUFV1_2; Protein -> CD44; Protein -> CBX1_2; Protein -> NDUFA5_2; Protein -> SYT7; Protein -> BIN1_4; Protein -> ATP5J2_1; Protein -> PLXNB1; Protein -> PADI2))) | Metabolite -> lysoPC a C20:3; Metabolite -> PC aa C36:6; Metabolite -> LCA; Metabolite -> lysoPC a C17:0; Metabolite -> HDCA; Metabolite -> PC aa C36:5 (RNA -> POLDIP3; RNA -> SARS; RNA -> LOC399715; RNA -> SMTN; RNA -> FRMPD2L2; RNA -> NDUFC2; RNA -> UHRF1; RNA -> FGF18; RNA -> SUNC1; RNA -> HS.213541; RNA -> PRKCH; RNA -> TAS2R10; RNA -> HS.570326; RNA -> DDIT4; RNA -> CLYBL; RNA -> STARD10; RNA -> ADNP2; RNA -> LOC650557; RNA -> MIB2; RNA -> LOC642521; RNA -> PLOD2; RNA -> TUBGCP5; RNA -> FAM59B; RNA -> GAL3ST3; RNA -> EIF2B2; RNA -> PEPD; RNA -> TOR1AIP2; RNA -> CHCHD6; RNA -> ISYNA1; RNA -> C5ORF15; RNA -> HS.583388; RNA -> NOLA2; RNA -> ACOT7; RNA -> TUBA1A; RNA -> GABBR1; RNA -> AQP11; RNA -> HS.145490; RNA -> FGF22; RNA -> SERPINF1; RNA -> ZNF215; RNA -> NAV2; RNA -> NFKB1; RNA -> AFF1; RNA -> AGFG2; RNA -> LOC651403; RNA -> CIB3; RNA -> POLR1E; RNA -> ASCL1; RNA -> NR1I2; RNA -> RFC2; RNA -> CSDE1; RNA -> INO80B; RNA -> PRG-3; RNA -> MED12; RNA -> HS.577965; RNA -> SOX21; RNA -> C11ORF76; RNA -> CDCA5; RNA -> HS.72367; RNA -> NTAN1; RNA -> LOC648364; RNA -> HOXC9; RNA -> UBE2D2; RNA -> LOC647096; RNA -> MPP2; RNA -> HS.567677; RNA -> HS.284464; RNA -> ZIC2; RNA -> HS.218036; RNA -> THOC5; RNA -> MTMR15; RNA -> AK3L1; RNA -> LRP8; RNA -> LOC646424; RNA -> ITPR2; RNA -> HIST1H2AH; RNA -> CLCNKA; RNA -> PRTG; RNA -> PVRL1) DNAm -> cg06481431; DNAm -> cg23449791; DNAm -> cg01553632; DNAm -> cg27609489; DNAm -> cg26366347; DNAm -> cg10678190; DNAm -> cg23044017; DNAm -> cg17949455; DNAm -> cg26361052; DNAm -> cg15830613; DNAm -> cg27059537; DNAm -> cg03383411; DNAm -> cg09298623; DNAm -> cg09798879; DNAm -> cg05505765; DNAm -> cg02081006; DNAm -> cg24433722; DNAm -> cg09329930; DNAm -> cg17512802; DNAm -> cg08439487; DNAm -> cg11348257; DNAm -> cg14757157; DNAm -> cg21463140; DNAm -> cg25932315; DNAm -> cg20777398; DNAm -> cg06145909; DNAm -> cg10269439; DNAm -> cg10679147; DNAm -> cg03139898; DNAm -> cg22682162; DNAm -> cg04515001; DNAm -> cg18050892; DNAm -> cg25691070; DNAm -> cg20638583; DNAm -> cg10215032; DNAm -> cg12652440; DNAm -> cg03907174; DNAm -> cg13602232; DNAm -> cg10409449; DNAm -> cg00934092; DNAm -> cg07470275; DNAm -> cg20659418; DNAm -> cg01021245; DNAm -> cg15927357; DNAm -> cg21438018; DNAm -> cg00425078; DNAm -> cg13704531; DNAm -> cg15899474; DNAm -> cg00056541; DNAm -> cg09114441; DNAm -> cg24675344; DNAm -> cg23288653; DNAm -> cg00500770; DNAm -> cg07604651; DNAm -> cg10607599; DNAm -> cg01527159; DNAm -> cg06636220; DNAm -> cg03713379; DNAm -> cg16514818; DNAm -> cg18225991; DNAm -> cg11946072; DNAm -> cg13657785; DNAm -> cg16588302; DNAm -> cg14186824; DNAm -> cg06177627; DNAm -> cg11361049; DNAm -> cg06238921; DNAm -> cg14531038; DNAm -> cg13715862; DNAm -> cg21772773; DNAm -> cg17918227; DNAm -> cg00711351; DNAm -> cg10128511; DNAm -> cg11491504; DNAm -> cg08108051; DNAm -> cg16293105; DNAm -> cg03339854; DNAm -> cg16465560; DNAm -> cg26672159; DNAm -> cg08459169; DNAm -> cg08573295; DNAm -> cg15643885; DNAm -> cg24319892; DNAm -> cg07850896; DNAm -> cg13162581; DNAm -> cg16622920; DNAm -> cg04144911; DNAm -> cg02919960; DNAm -> cg24673043; DNAm -> cg12695615; DNAm -> cg20644084; DNAm -> cg14872580 Protein -> C9orf16_2; Protein -> BIN1_9; Protein -> BIN1_7; Protein -> PIK3R1_1; Protein -> AP2B1_2; Protein -> SNAP29; Protein -> ELMO1_2; Protein -> MLF2_2; Protein -> SYN1 | Metabolite -> Glu; Metabolite -> Ala; Metabolite -> TwMCA; Metabolite -> Val; Metabolite -> Creatinine; Metabolite -> lysoPC a C RNA -> LOC731950; RNA -> MRPS16; RNA -> RFWD2; RNA -> SNORD56; RNA -> HS.581083; RNA -> HS.583637; RNA -> TBX20; RNA -> HS.156241; RNA -> HS.481464; RNA -> LPIN2; RNA -> UBAP1; RNA -> BRD9; RNA -> LAMA3; RNA -> CLCNKA; RNA -> HS.564742; RNA -> GPKOW; RNA -> HS.145476; RNA -> ARPC5; RNA -> WSB2; RNA -> BCL2L13; RNA -> WBP1; RNA -> HDHD2; RNA -> TOMM40L; RNA -> MANBAL; RNA -> NRSN2; RNA -> GCDH; RNA -> ABCF2; RNA -> DNAJC5B; RNA -> ACSL4; RNA -> C8ORF22; RNA -> HS.545454; RNA -> FIBP; RNA -> PYCRL; RNA -> GSTZ1; RNA -> PARD6G; RNA -> TRIAP1; RNA -> HS.294603; RNA -> JOSD1; RNA -> SDCCAG3; RNA -> CST1; RNA -> SPTBN4; RNA -> BEAN; RNA -> S100A13; RNA -> GABARAP; RNA -> HS.539959 DNAm -> cg06071660; DNAm -> cg19961277; DNAm -> cg27567587; DNAm -> cg16306508; DNAm -> cg21356750; DNAm -> cg14023762; DNAm -> cg05937687; DNAm -> cg03661929; DNAm -> cg03563694; DNAm -> cg14191675; DNAm -> cg10384134; DNAm -> cg04414459; DNAm -> cg12756244; DNAm -> cg09765463; DNAm -> cg19645248; DNAm -> cg05313918; DNAm -> cg21201272; DNAm -> cg03022609; DNAm -> cg03284175; DNAm -> cg00943950; DNAm -> cg04112626; DNAm -> cg08317267; DNAm -> cg22758189; DNAm -> cg16641000; DNAm -> cg19192649; DNAm -> cg08169778; DNAm -> cg20735988; DNAm -> cg10701733; DNAm -> cg07312485; DNAm -> cg15260780; DNAm -> cg06688409; DNAm -> cg09339907; DNAm -> cg20863107; DNAm -> cg12071888; DNAm -> cg19598875; DNAm -> cg19124521; DNAm -> cg07402261; DNAm -> cg06704539; DNAm -> cg00397144; DNAm -> cg12353428; DNAm -> cg21276022; DNAm -> cg08213351; DNAm -> cg14488256; DNAm -> cg07529658; DNAm -> cg05247112; DNAm -> cg23333911; DNAm -> cg00632861; DNAm -> cg24778130; DNAm -> cg10555010; DNAm -> cg00569056; DNAm -> cg03205103; DNAm -> cg13602813; DNAm -> cg25784395; DNAm -> cg21858823; DNAm -> cg24674754; DNAm -> cg12207450; DNAm -> cg27428418; DNAm -> cg00124774; DNAm -> cg24034106; DNAm -> cg10736063; DNAm -> cg15824316; DNAm -> cg19497501; DNAm -> cg26149167; DNAm -> cg14889329; DNAm -> cg26436829; DNAm -> cg14983606; DNAm -> cg07201319; DNAm -> cg21453656; DNAm -> cg25407736; DNAm -> cg16878027; DNAm -> cg04570362; DNAm -> cg03333286; DNAm -> cg20808078; DNAm -> cg07492518; DNAm -> cg02990705; DNAm -> cg17337483; DNAm -> cg24854787; DNAm -> cg27008565; DNAm -> cg15211499; DNAm -> cg24777572; DNAm -> cg25367890; DNAm -> cg26777809; DNAm -> cg22253078; DNAm -> cg14874936; DNAm -> cg02491199; DNAm -> cg03563083; DNAm -> cg07509323; DNAm -> cg24928681; DNAm -> cg09946915; DNAm -> cg05562381 Protein -> CLTA_1; Protein -> LDHA_2; Protein -> BIN1_3; Protein -> STX12; Protein -> STXBP1_3; Protein -> SNAP25_6 |

**S.Table 3.** Mean F-values in percentages (95%CIs) for each omic modality by AD subtypes.

| **AD subtype** | **Epigenomic** | **Transcriptomic** | **Proteomic** | **Metabolomic** |
| --- | --- | --- | --- | --- |
|  | **Mean (95%CI)** | **Mean (95%CI)** | **Mean (95%CI)** | **Mean (95%CI)** |
| **1** | 8.8 (8.3-9.4) | 14.1 (12.8-15.5) | 6.9 (5.7-8.0) | 41.5 (35.9-47.1) |
| **2** | 22.4 (20.5-24.3) | 34.6 (31.5–37.6) | 15.1 (11.8-18.3) | 81.2 (69.7-92.6) |
| **3** | 12.3 (11.1-13.4) | 15.5 (14.1-17.1) | 7.1 (5.7-8.2) | 63.7 (39.6-87.8) |

**Note.** Higher f-value show stronger differences from the NCI.

**S.Table 4.** Post-hoc comparisons using Bonferroni’s method for inter-subtype differences on the F-value which represents significantly different features from the no cognitive impairment group.

|  | **Subtype-subtype comparisons** | **Difference in means on the F-value (%)** | **95% CI** | | ***p*** |
| --- | --- | --- | --- | --- | --- |
| **Metabolites** | **1 – 2** | **39.7** | **21.6** | **57.7** | **<0.001** |
|  | **1 – 3** | **22.3** | **4.2** | **40.3** | **0.013** |
|  | 2 – 3 | 17.5 | -6.2 | 41.1 | .184 |
| **RNAseq** | **1 – 2** | **20.6** | **17.0** | **24.2** | **<0.001** |
|  | 1 – 3 | 1.6 | 2.6 | 5.7 | 1.00 |
|  | **2 – 3** | **19.0** | **14.9** | **23.2** | **<0.001** |
| **CpGs** | **1 – 2** | **13.9** | **11.9** | **15.8** | **<0.001** |
|  | **1 – 3** | **3.4** | **1.5** | **5.4** | **<0.001** |
|  | **2 – 3** | **10.5** | **8.4** | **12.5** | **<0.001** |
| **Proteins** | **1 – 2** | **8.2** | **5.2** | **11.3** | **<0.001** |
|  | 1 – 3 | 0.09 | -3.4 | 3.6 | 1.00 |
|  | **2 – 3** | **8.1** | **4.4** | **11.8** | **<0.001** |

**Note.** The largest inter-subtype differential F-values relative to NCI were consistently seen between AD1 and AD2 with differences in F-values ranging from 8% for proteomic alterations to 40% for metabolomic differences with AD2 consistently showing higher differential values from AD1. The smallest inter-subtype differences were observed for AD1 and AD3 with differences ranging from only 0.1% for proteomics differences to 22% for metabolomic differences differentiating them from NCI. Indeed, no significant differences were observed between AD1 and AD3 for proportions of RNA and proteomic alterations.

**S.Table5.** Number of pathologies stratified by no cognitive impairment (NCI) and AD subtypes.

| Frequency of pathologies | NCI | AD1 | AD2 | AD3 |
| --- | --- | --- | --- | --- |
|  | N (%) | N (%) | N (%) | N (%) |
| 0 | 34 (13.4) | 1 (0.6) | 6 (3.3) | 6 (3.3) |
| 1 | 65 (25.7) | 25 (14.5) | 19 (10.6) | 19 (12.4) |
| 2 | 70 (27.7) | 34 (19.7) | 32 (17.8) | 27 (17.6) |
| 3 | 48 (19.0) | 37 (21.4) | 36 (20.0) | 30 (19.6) |
| 4 | 21 (8.3) | 36 (20.8) | 39 (21.7) | 33 (21.6) |
| 5 | 13 (5.1) | 28 (16.2) | 24 (13.3) | 27 (17.6) |
| 6 | 1 (0.4) | 6 (3.5) | 16 (8.9) | 10 (6.5) |
| 7 | 1 (0.4) | 6 (3.5) | 7 (3.9) | 2 (1.3) |
| 8 | 0 | 0 | 0 | 0 |
| 9 | 0 | 0 | 1 (0.6) | 0 |

**Figures**

**sFigure 1.** **Box and whisker plots of inter-subtype molecular differences.** Each omic data feature shows a comparison amongst the three AD subtypes. Each plot corresponds to the percent of significantly different features in percent F-value for each omic modality, based on ANOVA tests with subtype as the grouping measure. The dots in each plot represent a feature within that omic modality; the top and bottom of the boxes represent quartiles 1 and 3, and the whiskers show the inter-quartile range. The middle horizontal line represents the mean percent of data features that are abnormal for that subtype.

**sFigure 2. Percentage of individuals with ADRD pathologic indices stratified by No cognitive impairment (background) and multi-omic brain molecular AD subtypes.** Pathologic indices include diagnosis of AD, non-AD neurodegenerative pathology (neocortical Lewy bodies, TPD-43, and hippocampal sclerosis), cerebral (gross and/or micro) infarcts, and cerebral vessel pathology (arteriosclerosis, atherosclerosis, and cerebral amyloid angiopathy). Different colored bars represent the percentage of individuals within the no cognitive impairment (NCI) group and each AD subtype and add up to 100% for each individual group.

*Note.* Blue bars = no cognitive impairment; Orange bars= AD1; Red bars=AD2; Purple bars=AD3.

**sFigure 3.** Differences in brain morphology between AD subtypes and the background (FWER *p*≤.05), adjusted for age at death, sex, race, education, postmortem scanning interval and location. Warm and cold colors indicate larger and smaller volumes relative to the background, respectively.

**sFigure 4.** **Polygenic risk score prediction of Pseudotime and AD subtypes.** **A.** Scatterplot displaying the association between genetic prediction as determined by an overall correlation between 802 polygenic risk scores and pseudotime. **B.** Bar plot showing overall polygenic risk score prediction of AD subtypes. The black dots represent the area under the receiver operating characteristic (ROC) curve with 95% confidence intervals (shaded rectangles). **C.** ROC curve that shows that sensitivity and specificity of polygenic risk scores in distinguishing amongst the groups. Higher sensitivity and specificity were achieved in genetic prediction distinguishing the no cognitive impairment, background group from the AD subtypes.
