# Supplementary material for: Associations of stable psychological traits with multi-omic subtypes of Alzheimer’s dementia
Source: Transl Psychiatry. 2026 Jun 21;16:443. doi: 10.1038/s41398-026-04195-z (PMC13530231; doi:10.1038/s41398-026-04195-z)
Supplement: Supplementary file 2 [file 41398_2026_4195_MOESM3_ESM.docx]

**eMethods**

**Associations of stable psychological traits with multi-omic subtypes of Alzheimer’s dementia**

**Zammit et al.**

Both cohorts (ROS and MAP) have identical study design and a large common core of data collection at the item level. The same trainer, supervisor and testers collect the data, and the brains go through the same neuropathologic and omic pipelines.

1. **Neuropathologic evaluation**

Briefly, brains were removed and weighed, and hemispheres were cut coronally using a Plexiglas^®^ jig into 1cm slabs. One hemisphere was fixed in 4% paraformaldehyde. After gross examination of both hemispheres, nine brain regions of interest (i.e. mid-frontal, mid-temporal, inferior parietal, anterior cingulate, entorhinal and hippocampal cortices, basal ganglia, thalamus, and mid-brain) were dissected from the 1cm slabs of fixed tissue and processed and embedded in paraffin. Sections (6mm) from the paraffin blocks were stained for assessment of pathology.

**AD and non-AD neurodegenerative pathologies.** For AD pathological diagnosis, a modified Bielschowsky silver stain was used to visualize neuritic plaques, diffuse plaques, and neurofibrillary tangles in five cortical areas (hippocampus, entorhinal, midfrontal, middle temporal, and inferior parietal). A board-certified neuropathologist or an expert neuroscientist, blinded to clinical data, determined the pathologic diagnosis of AD, defined as intermediate to high likelihood of AD according to NIA Reagan criteria^51^. The presence of neocortical Lewy body pathology was determined using antibodies to a-synuclein^52^. The presence of hippocampal sclerosis was determined using H&E stain^53^. The presence of TDP-43 cytoplasmic inclusions was determined using antibodies to phosphorylated TDP-43; TDP-43 distribution was grouped into 3 stages (stage 1, localized to amygdala; stage 2, extension to hippocampus or entorhinal cortex; stage 3, extension to the neocortex) and TDP-43 was considered present if positive for stages 2 or 3^54^.

**Vascular pathologies.** Gross chronic infarcts were identified by visually examining slabs and pictures from both brain hemispheres and confirmed histologically, and microinfarcts (chronic) were identified under microscopy using hematoxylin and eosin stain^49^. Moderate to severe arteriolosclerosis was detected by use of hematoxylin and eosin stained section of the anterior basal ganglia^55^. Moderate to severe atherosclerosis was detected by visual inspection of vessels in the Circle of Wilis^55^. Moderate to severe cerebral amyloid angiopathy was detected using amyloid-B immunostaining in four cortical regions^55, 56^.

2. **Postmortem Imaging**

These T2 images were later corrected for N4 bias field inhomogeneity^51^ and non-linearly registered to an in-house postmortem hemispheric template^52^ using Advanced Normalization Tools (ANTs)^53^. This registration was used to compute voxel-wise Jacobian determinant maps, which we then log-transformed and smoothed with a 4mm FWHM Gaussian kernel. Higher values in these deformation maps indicated large volumes that were contracted to fit the template, whereas lower values indicated smaller volumes that were expanded to the template.

3. **Psychometric characteristics of the psychological measures**

**Neuroticism.** Neuroticism is an indicator of proneness to psychological distress. Participants rated agreement with each item (e.g. “I often feel inferior to others”), on a 5-point Likert scale ranging from 0 (strongly disagree) to 4 (strongly agree). The total score in participants with the 12-item scale was computed by summing item scores to yield a total score that ranged from 0 to 48, with higher scores indicating more proneness to psychological distress. The total score of those with the 6-item version was computed by adding the item scores and then multiplying the sum by two to make the score comparable to the standard 12-item version. The correlation of the 6-item version with the standard 12-item scale is 0.90, as previously reported^22^, supporting the validity of the 6-item scale. Internal consistency reliability as determined by the Cronbach coefficient alpha was 0.8, which indicates adequate internal consistency.

**Depressive symptoms.** The CES-D is widely used in epidemiologic studies of older persons, and the reliability of this version with its original version has been previously reported^38^. Each item represented a symptom, and participants indicated whether they experienced the symptom during the past week. The score was the total number of symptoms experienced.

**Loneliness.** Participants rated agreement with each item (e.g. “I experience a general sense of loneliness.”) on a 5-point Likert scale ranging from 1 (strongly disagree) to 5 (strongly agree). These five items were selected to explicitly measure the psychological and feelings of isolation. The original version of this scale has been shown to be internally consistent^42, 43^.

**Purpose in life.** Participants rated agreement with each item (e.g. “I enjoy making plans for the future and working them to a reality.”) on a 5-point Likert scale ranging from 1 (strongly disagree) to 5 (strongly agree). The total score was then averaged to yield a total score, with higher scores indicating higher purpose in life.

4. **Testing replicability of derived pseudotime and subtypes across two independent samples**

We used two independent samples to test the generalizability of the Pseudotime using post-mortem bulk transcriptomic and proteomic data.

The first independent sample came from ROSMAP. Here, we analyzed post-mortem transcriptomic and proteomic data derived from the DLPFC of 223 ROSMAP participants who were not included into no cognitive impairment, mild cognitive impairment, and AD dementia categories. As in the original analysis, omics features (gene abundance and protein concentration) were adjusted for age, sex, education, postmortem interval (PMI) in hours, sample pH, RNA integrity number, and batch number when applicable, using robust additive linear models.

The second independent sample came from human brains that were assessed from the Mount Sinai/JJ Peters VA Medical Center Brain Bank (MSBB-Mount Sinai NIH Neurobiobank) cohort, which holds over 2,040 well-characterized brains).^41, 42^ This cohort was assembled after applying stringent inclusion/exclusion criteria and represents the full spectrum of cognitive and neuropathological disease severity in the absence of discernable non-AD neuropathology. The Clinical dementia rating scale (CDR) was conducted for assessment of dementia and cognitive dementia and cognitive status. All neuropsychological, diagnostic, and autopsy protocols used for the MSBB study were approved by the Mount Sinai and JJ Peters VA Medical Center Institutional Review Boards.

For MSBB, we analyzed post-mortem bulk transcriptomic and proteomic data from the parahippocampal gyrus of 118 participants (synapse ID: syn3159438). We assigned participants based on CDR classification (NCI CDR=0, 20%; questionable dementia CDR=0.5, 21%; mild, moderate and severe dementia CDR≥1, 59%). The omics data were preprocessed by adjusting for age at death, sex, race, post-mortem interval (PMI) in hours, RIN (for RNAseq), exonic rate (for RNA seq) and batch. The adjusted MSBB omics data were then aligned with the bulk transcriptomics and proteomic data from the DLPFC in ROSMAP (concatenating genes’ expression and proteins’ concentration across cohorts). Thus, we restricted the analysis to common genes and proteins within those previously used in the ROSMAP mcTI analysis. Cohort effects were corrected for each gene/protein using robust linear regression.

**Projection of Pseudotime.** A ten-fold cross-validated linear regression model was trained in the primarily used ROSMAP dataset, with gene and protein expression as predictors of AD dementia pseudotime. Resulting model estimates were applied to the ROSMAP replication dataset and separately MSBB replication dataset (obtaining AD dementia pseudotime projections for these two additional groups of participants). ANOVA tests with 1000 randomized permutations were used to test the association between the projected pseudotime and global cognition in the ROSMAP replication dataset and with dementia severity in the MSBB replication dataset.

**Projection of subtypes.** ROSMAP participants from the discovery sample were used to train 10-fold cross-validated linear classifiers predicting subtype membership in the ROSMAP taxonomy. Classifier parameters were estimated on the transcriptomic and proteomic layers from the discovery ROSMAP participants and applied to the additional 223 ROSMAP participants with the same omic features. These analyses yielded a transferred classification of independent ROSMAP participants into three discovery ROSMAP-derived subtypes. We then employed Normalized mutual information (nMI)^60^ to compare the subtype solutions on the common 223 participants. The significance of this value was evaluated using 10,000-label permutation tests, generating empirical null distributions for each metric.
